# Solitary Fibrous Tumor With Doege–Potter Syndrome Successfully Treated With Preoperative Transcatheter Arterial Embolization and Complete Excision; A Case Report

**DOI:** 10.1002/iju5.70107

**Published:** 2025-10-31

**Authors:** Rei Narimatsu, Ryuji Matsumoto, Haruka Miyata, Takahiro Osawa, Daisuke Abo, Kento Wakabayashi, Utano Tomaru, Hiraku Kameda, Izumi Fukuda, Takashige Abe

**Affiliations:** ^1^ Department of Urology Hokkaido University Hospital Sapporo Japan; ^2^ Department of Diagnostic and Interventional Radiology Hokkaido University Hospital Sapporo Japan; ^3^ Department of Surgical Pathology Hokkaido University Hospital Sapporo Japan; ^4^ Department of Rheumatology, Endocrinology, and Nephrology, Faculty of Medicine and Graduate School of Medicine Hokkaido University Sapporo Japan; ^5^ Department of Endocrinology, Metabolism and Nephrology, Graduate School of Medicine Nippon Medical School Tokyo Japan

**Keywords:** Doege–Potter syndrome, solitary fibrous tumor, transcatheter arterial embolization

## Abstract

**Introduction:**

Doege–Potter syndrome (DPS) is a rare paraneoplastic phenomenon of severe hypoglycemia associated with solitary fibrous tumors (SFT). We report a case of a giant pelvic SFT with DPS, which was managed with preoperative arterial embolization and complete excision.

**Case Presentation:**

This report describes the case of a 77‐year‐old patient with persistent hypoglycemia and a giant pelvic mass. He required continuous total parenteral nutrition (TPN) for severe hypoglycemia. CT showed a giant hypervascular mass (20 × 18 × 15 cm) in the pelvic space. Tumor biopsy showed SFT. To avoid intraoperative brisk bleeding, transcatheter arterial embolization (TAE) of the main feeders was performed 1 day before surgery. The tumor was completely resected via midline abdominal incision. Hypoglycemia resolved postoperatively. He was recurrence‐free for 11 months after surgery.

**Conclusion:**

The combination of preoperative TAE and surgical resection appears to be an effective therapeutic strategy for DPS‐associated SFT.

**Summary**

- We report a case of a giant pelvic solitary fibrous tumor (SFT) presenting with severe hypoglycemia, along with Doege–Potter syndrome.
- To avoid the risk of intraoperative brisk bleeding, preoperative transcatheter arterial embolization (TAE) was performed to occlude the primary feeding vessels.
- This approach enabled the safer surgical excision of the tumor without an extensive amount of blood loss, leading to the immediate recovery from hypoglycemia.
- Accordingly, preoperative TAE represents a valuable and efficacious adjunctive strategy that facilitates the complete resection of large pelvic SFT.

## 1 Introduction and Background

Solitary fibrous tumor (SFT) is a rare mesenchymal neoplasm of fibroblastic origin. In fewer than 5% of cases, it is associated with refractory hypoglycemia, known as Doege–Potter syndrome (DPS) [1, 2]. This paraneoplastic hypoglycemia results from tumor‐derived secretion of high‐molecular‐weight insulin‐like growth factor 2 (IGF‐II) [3].

Since its first report in 1930, the management of DPS has remained non‐systematic due to its rarity [4, 5]. Herein, we present a case of pelvic SFT with DPS successfully managed with preoperative transcatheter arterial embolization (TAE) and complete excision.

## 2 Case Presentation

**FIGURE 1:**
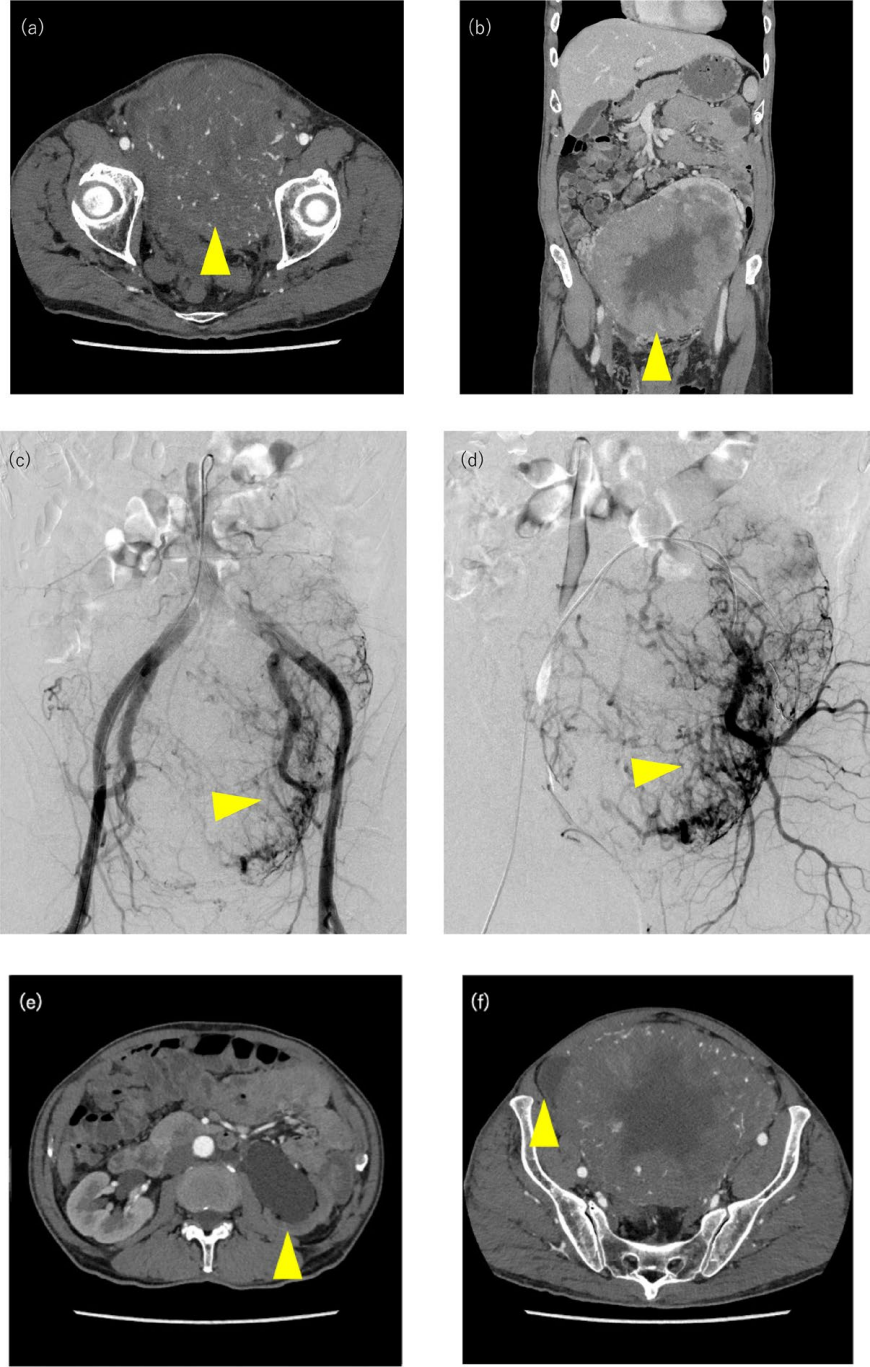
(a) Contrast‐enhanced CT showed enhancement of the tumor in a heterogeneous pattern. (b) A giant mass occupied in the pelvic cavity. (c, d) branches of the left internal iliac artery were the main feeders. (e) Left hydronephrosis developed due to ureteral obstruction. (f) Bladder was completely displaced to the right side.

A 77‐year‐old man was emergently transported due to loss of consciousness. Examination revealed hypoglycemia and an intrapelvic mass. He required continuous total parenteral nutrition (TPN) due to severe hypoglycemia. His medical history was notable only for hypertension, and he had no history of diabetes mellitus or previous surgery. Computed Tomography (CT) showed a giant hypervascular mass in the pelvic space (Figure 1a,b); branches of the left internal iliac artery were the main feeders (Figure 1c,d). The bladder was completely displaced to the right side, and left hydronephrosis also developed due to ureteral obstruction (Figure 1e,f). Laboratory examination, including tumor markers, revealed no abnormalities except for hypoglycemia (plasma glucose 26 mg/dL) and suppressed serum levels of insulin (< 1.6 μU/mL; normal range, 5.0–10.0 μU/mL) and C‐peptide (0.28 ng/mL; normal range, 0.78–5.19 ng/mL) upon endocrinological evaluation. Tumor needle biopsy revealed a diagnosis consistent with SFT. The presence of a giant pelvic mass in conjunction with recurrent hypoglycemic episodes raised a strong clinical suspicion for DPS. To avoid intraoperative brisk bleeding, TAE of the main feeders was performed using a gelatin sponge 1 day before surgery. The tumor was completely resected via midline abdominal incision from xiphoid process to pubic tubercle. During the surgery, the left ureter was accidentally injured. Although the bladder and left kidney were preserved, the tumor was firmly adhered to the left ureter, requiring a partial urethrectomy and subsequent left ureteroneocystostomy via extravesical approach. The operative time was 681 min, and the total blood loss was 1680 mL. Twelve units of packed red blood cells (RBC) were transfused intraoperatively. Hypoglycemia resolved postsurgery. Although he developed paralytic ileus and venous thrombus of the external iliac vein (Clavian‐Dindo grade II), they were conservatively managed.

**FIGURE 2:**
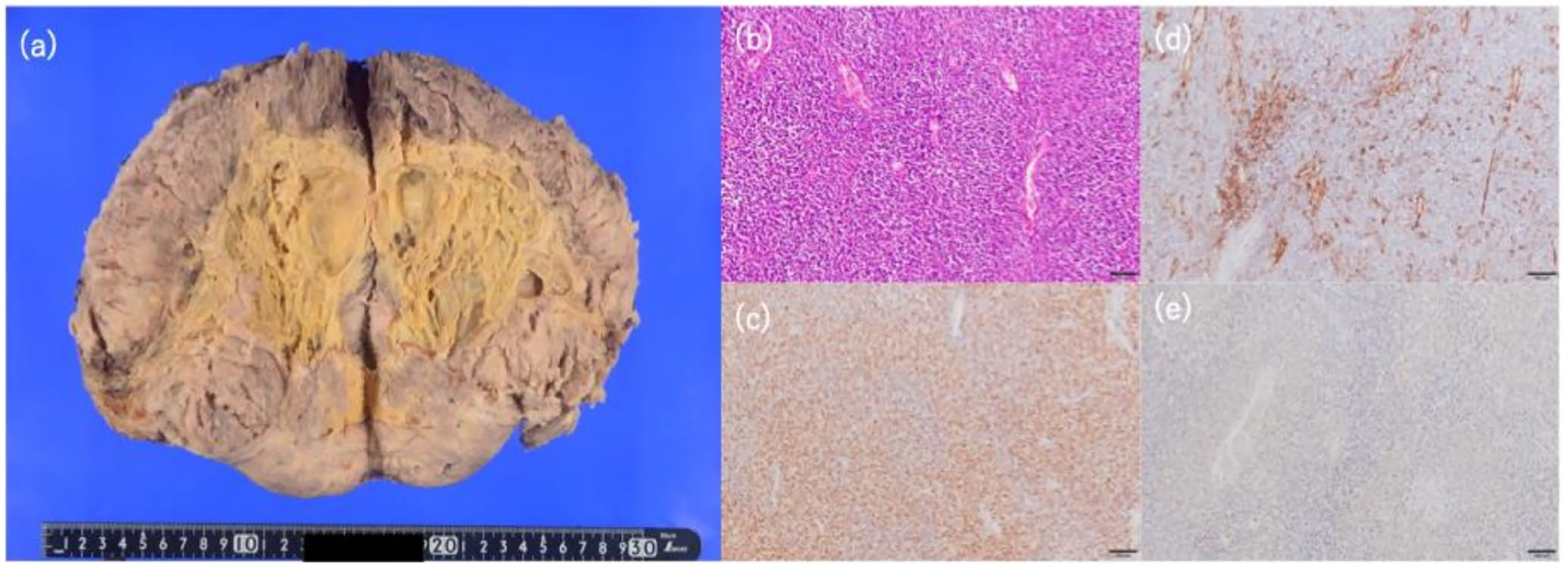
Pathological image. (a) macroscopic appearance (20 × 18 × 15 cm). (b) 10× hematoxylin and eosin staining. (c) 10× positive STAT6. (d) 10× positive CD34. (e) 20× weakly positive for IGF‐II on immunohistochemical examination.

**FIGURE 3:**
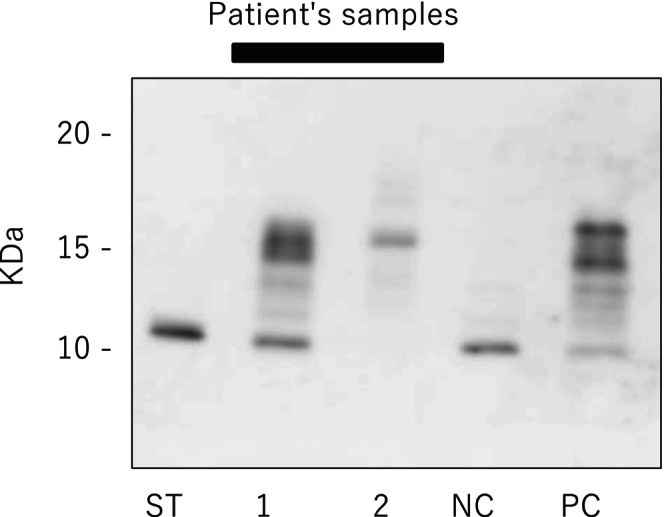
Western immunoblot analysis of both the serum and the biopsy specimen demonstrated the presence of high molecular weight IGF‐II. The dence band at around 15 kDa (high molecular weight IGF‐II) in the preoperative serum (lane 1). The tumor tissue also had an IGF‐II band (lane 2). NC, negative control; PC, positive control; ST, standard of this IGF‐II antybody.

The patient was discharged from the hospital on the 23rd postoperative day. Histopathology confirmed SFT, with immunohistochemical staining positive for CD34 and STAT6, and weakly positive for IGF‐II (Figure 2). Western immunoblot analysis of both preoperative serum and needle biopsy specimens demonstrated the presence of high molecular weight IGF‐II (Figure 3), further supporting the diagnosis of DPS associated with SFT. The tumor was classified as high‐risk (7 points) by the modified Demicco criteria based on age, size (20 cm), mitotic activity (4/10 high‐power fields), and necrosis. Due to the lack of robust evidence of adjuvant therapy, advanced age, and patient preference, he was managed conservatively with close observation and follow‐up. At follow‐up, CT showed complete surgical tumor resection and no evidence of recurrence in the 11 months after the surgery.

## 3 Discussion

DPS represents a rare paraneoplastic phenomenon linked to SFT, distinguished by tumor‐induced hypoglycemia resulting from excessive secretion of IGF‐II. This report presents a case of pelvic SFT accompanied by DPS. The gold standard of treatment is surgery, but if surgery is not possible, radiation therapy, chemotherapy, or immunotherapy has also been reported to be effective [5, 6]. In the present case, the patient had no significant comorbidities and was deemed physiologically fit for surgery; therefore, curative resection was considered feasible and was successfully performed.

Importantly, SFTs associated with DPS often exhibit pronounced hypervascularity, a feature that substantially elevates the risk of intraoperative hemorrhage. This concern is accentuated in pelvic neoplasms, where intricate vascular architecture further complicates intraoperative hemostatic control. Table 1, which consolidates previously documented cases of pelvic DPS [7, 8, 9, 10, 11, 12, 13, 14, 15, 16], shows a median intraoperative blood loss of 1620 mL (range: 335–8300 mL). Furthermore, recent literature indicates that certain cases of intra‐abdominal SFT with a DPS component have been associated with massive intraoperative hemorrhage exceeding 10,000 mL [6]. These findings emphasize the critical need for comprehensive preoperative planning.

**TABLE 1:** Literature review of all documented instances of pelvic solitary fibrous tumor associated with Dorge–Potter syndrome.

Author	Age/sex	Location	Size	Treatment	Blood loss	Urinary and fecal diversion	TAE	Follow‐up
Wakami et al. [7]	78 F	Uterin	23 cm	Local resection of the uterine tumor	n/a	n/a	n/a	No reccurence at 2 years
Hosaka et al. [8]	62 F	Pelvic cavity	14 cm	Intra‐arterial chemothrapy, conccurrent radiotherapy	n/a	n/a	n/a	No metastases at 5 years since diagnosis. 2 years since chemo, no further hypoglycaemia
Nirosshan et al. [9]	70 F	Pelvic cavity	20 × 13 × 14 cm	Oral administration of prednisolone due to refuse surgical resection	n/a	n/a	n/a	Titration of prednisone dose to 5 mg daily at 1‐year follow‐up without further hypoglycemia
Chen et al. [10]	37 F	Broad ligament	16 × 15 × 15 cm	Surgical resection	5000 mL	n/a	n/a	Reccerence at 43 months post operation
Wada et al. [11]	72 M	Pelvic cavity	15 × 8 × 8 cm	Surgical resection	1410 mL	n/a	n/a	No reccurence at 1 year
Yuza et al. [12]	46 F	Pelvic cavity	17 cm	Embolization and resection	335 mL	n/a	2 days prior to surgery	No reccurence at 2 years
Santos et al. [13]	72 F	Ovary	10 × 6.9 cm	Surgical resection	n/a	n/a	n/a	No reccurence at 3 months
Kiely et al. [14]	62 F	Broad ligament	18 cm	Total abdominal hysterectomy	8300 mL	n/a	n/a	No reccurence at 6 months
Deguchi et al. [15]	70 F	Uterin	19 cm	Tumor resection with combined resection of the rectum (low anterior resection) and left ureter (accompanied with uretero‐cysto‐neostomy)	1560 mL	Resection of the rectum and left uretero‐cysto‐neostomy	n/a	Reccurence at 5 months after surgery
Mao et al. [16]	82 F	Pelvic cavity	12 × 10 × 8 cm	Surgical resection	n/a	n/a	n/a	n/a
The present case	77 M	Pelvic cavity	20 × 18 × 15 cm	Tumor resection and left Uretero‐cysto‐neostomy	1680 mL	Left uretero‐cysto‐neostomy	1 day prior to surgery	No reccurence at 11 months

In light of these findings, preoperative TAE may serve as a valuable adjunctive intervention to attenuate intraoperative blood loss. Although specific guidelines regarding the optimal timing of TAE in SFT cases are lacking, relevant evidence from analogous conditions offers informative parallels. For instance, in the management of uterine fibroids—another pelvic hypervascular tumor—TAE administered within 24 h preoperatively has demonstrated efficacy in reducing intraoperative hemorrhage and enhancing surgical safety [17]. Conversely, studies on spinal tumors have shown no statistically significant differences in blood loss between cohorts receiving TAE within 24 h versus 24–48 h prior to surgery [18]. These heterogeneous findings underscore the potential utility of TAE in the perioperative management of hypervascular SFTs, particularly those complicated by DPS. However, the determination of optimal timing remains inconclusive.

Additionally, according to Demicco et al. [19], the modified Demicco risk stratification model shows a significant prognostic value, particularly in predicting recurrence‐free survival (RFS) after surgical resection of SFT. In their multivariate Cox proportional hazards analysis, high‐risk tumors showed a markedly increased risk of recurrence compared to low‐risk tumors (HR 10.14, *p* = 0.001). These findings support the prognostic utility of the modified Demicco criteria, particularly in identifying patients at elevated risk for early recurrence following surgical resection. In the present case, the patient's total risk score was 7, placing him in the high‐risk category based on the modified Demicco model. Given the strong association between high‐risk classification and poor RFS demonstrated in previous studies, adjuvant therapy was considered to reduce the likelihood of recurrence. Radiation therapy or a chemotherapy regimen (doxorubicin, ifosfamide, and gemcitabine) was suggested as a potential adjuvant therapy [6]. However, after the thorough discussion of the potential benefits and risks, adjuvant treatment was ultimately not pursued, primarily due to the patient's advanced age and his preference. Accordingly, a conservative approach with close observation was adopted for postoperative management. Continued accumulation of case‐based evidence and prospective analysis are imperative to establish evidence‐based perioperative protocols and optimize clinical outcomes in this rare yet complex oncologic entity.

## 4 Conclusion

The combination of preoperative TAE and surgical resection appears to be an effective therapeutic strategy for DPS‐associated SFT, particularly in terms of intraoperative brisk bleeding and overall surgical safety. Further accumulation of clinical cases and long‐term follow‐up is necessary to facilitate the standardization of treatment protocols.

## Consent

We obtained informed consent from the patient.

## Conflicts of Interest

The authors declare no conflicts of interest.
